# A novel acrylic resin palatal device contaminated with *Candida albicans* biofilm for denture stomatitis induction in Wistar rats

**DOI:** 10.1590/1678-7757-2020-0865

**Published:** 2021-04-19

**Authors:** Gustavo Simão MORAES, Thaís ALBACH, Isabella Esser RAMOS, Mariana Gomes KOPACHESKI, Victoria Schlumberger CACHOEIRA, Carolina Yoshi Campos SUGIO, César Augusto Galvão ARRAIS, Karin Hermana NEPPELENBROEK, Vanessa Migliorini URBAN

**Affiliations:** 1 Universidade Estadual de Ponta Grossa Departamento de Odontologia Ponta GrossaParaná Brasil Universidade Estadual de Ponta Grossa, Departamento de Odontologia, Ponta Grossa, Paraná, Brasil.; 2 Universidade de São Paulo Faculdade de Odontologia de Bauru BauruSão Paulo Brasil Universidade de São Paulo, Faculdade de Odontologia de Bauru, Bauru, São Paulo, Brasil.

**Keywords:** Denture stomatitis, Candida albicans, Animal model, Wistar rats

## Abstract

Denture stomatitis is the most frequent oral lesion in removable prosthesis wearers, with high recurrence rates and a complex treatment.

**Objective:**

This study describes a protocol to obtain and to contaminate a palatal device with *Candida albicans* biofilm that could be used for an animal model of denture stomatitis.

**Methodology:**

Acrylic resin devices (N=41) were obtained from impressions of the palates of Wistar rats with individual trays and polyether. The efficacy of microwave irradiation (MW), ultraviolet light (UV), or ultrasonic bath (US) was assessed by colony viability and spectrophotometric analyses (n=5) in order to select the most appropriate method for sterilizing the devices. Then, different devices (n=5) were contaminated with *C. albicans* and evaluated by CFU/mL determination, scanning electron microscopy, and laser confocal microscopy. Device stabilization was assessed with either autopolymerizing acrylic resins or a self-adhesive resin cement (n=2). The spectrophotometric data were analyzed by one-way ANOVA followed by the Tukey’s HSD post-hoc test (α=0.05).

**Results:**

MW was the only method capable of sterilizing the devices, and the contamination protocol developed a mature and viable *C. albicans* biofilm (~1.2 x 10^6^ CFU/mL). The self-adhesive resin cement was the best stabilization material.

**Conclusions:**

This acrylic resin palatal device was designed to be similar to the clinical situation of contaminated prostheses, with easy manufacturing and handling, effective stabilization, and satisfactory contamination. Thus, the acrylic device can be a valuable tool in the development of denture stomatitis in rats.

## Introduction

Oral candidiasis is one of the most common oral fungal infections,^1,2^ and it is called denture stomatitis when associated with the wearing of removable dental prosthesis. The etiology of denture stomatitis includes local and systemic factors.^3^ Despite the multifactorial etiology, its main cause is the infection by *Candida* spp., especially by *Candida albicans*.^4^

Treating denture stomatitis is challenging. It involves the improvement of oral and denture hygiene,^5^ regular denture immersion in antiseptic or disinfectant products,^3,5-8^ relining or replacing the prostheses, and the topical and/or systemic administration of antifungal drugs.^3,7^ However, the treatment effectiveness can be impaired by the medication cost and its unpleasant taste,^9^ the poor compliance with the treatment,^7^ and especially the mucosa reinfection due to the use of a contaminated prosthesis.^7,10^ To overcome these limitations, other therapeutic modalities have been proposed, including denture microwave disinfection,^11^ photodynamic therapy,^10^ and the incorporation of antifungal agents in denture adhesives^12^ and soft lining materials.^13^

Prior to clinical trials, it is essential to ensure that any alternative treatment is safe and effective. Consequently, an *in vivo* model of denture stomatitis is necessary. Numerous protocols to induce oral candidiasis in animals have been reported. One option is to inoculate suspensions of *C. albicans* into the oral cavities of the animals. Although this method can produce signs of oral candidiasis at some sites of the oral cavity,^14-17^ it is not capable of promoting clinical and histological changes in the palate without being associated with an acrylic resin device.^18^

Some authors tried to induce denture stomatitis in rats by fitting acrylic resin devices into their palates. Rather than being contaminated with *C. albicans*, these devices served only as a container for the candida suspension, which is directly inoculated on the palate and/or placed in the internal surface of the device immediately before its cementation.^18-22^ These devices require laborious fabrication,^19^ do not mimic the clinical conditions of a contaminated prosthesis, and might impair food intake.^23^ This study describes a protocol to obtain and to contaminate a palatal device with *Candida albicans* biofilm that closely reproduces the clinical condition of contaminated dentures and that could be used for an animal model of denture stomatitis.

## Methodology

### Animals

This study was performed in compliance with the Guiding Principles for the Care and Use of Animals and the ARRIVE guidelines and it was approved by the Ethics Committee on Animal Use (CEUA) of the State University of Ponta Grossa (UEPG, protocol number: 048/2016). Six male Wistar rats (*Rattus novergicus albinus*), weighing from 250 to 300 g, were kept in plastic cages in a temperature-controlled room (22±2°C) under a 12-hour light/dark cycle with pellet food and water *ad libitum*. Throughout the experiments, weight, general health, and well-being of the animals were observed, and, if any animal was to become severely ill or debilitated, it would be euthanized.

The intraoral procedures were performed under general anesthesia by intraperitoneal injection with 90 mg/kg of 10% ketamine (Ketalex; Rhobifarma Indústria Farmacêutica Ltda., Hortolândia, SP, Brazil) and 10 mg/kg of 2% xylazine (Xilazin; Syntec do Brasil Ltda., Santana de Parnaíba, SP, Brazil). The anesthetized rats were placed on a stabilizing bed, and their mouths were kept open by using loops connected to their maxillary and mandibular incisors.^23^ Following the procedures, the animals were placed in a warm environment and were monitored until they recovered from anesthesia and then were returned to their cages and to the animal facility.

### Palatal acrylic resin device fabrication and sterilization

**Figure 1:**
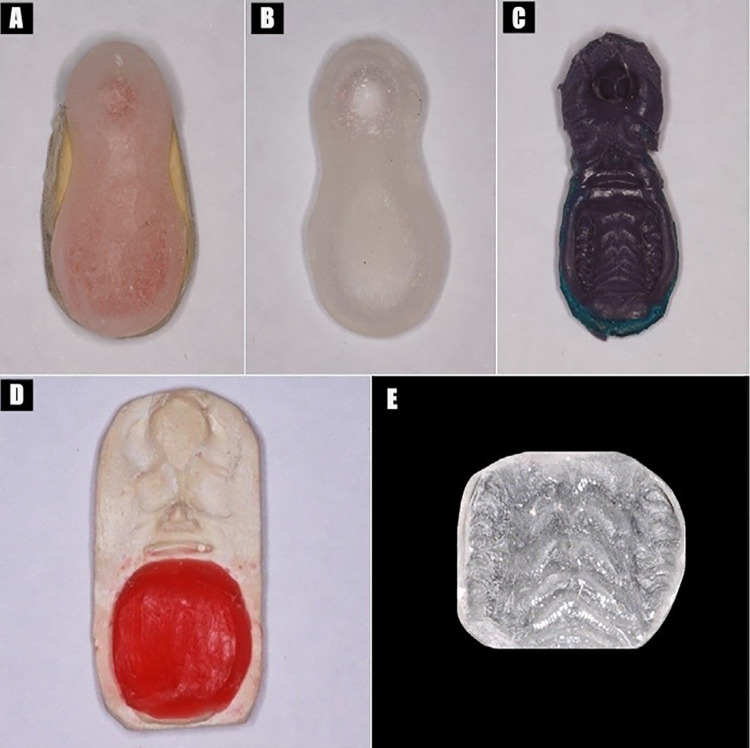
Palatal device fabrication. A: a tray relief area on a previously determined extension of the preliminary casts; B: individual tray after polishing; C: palatal impression; D: waxed type IV stone working casts; E: intraoral device after polishing

This intraoral acrylic resin device is an improved version of a previously described design.^23^ Initially, impressions of a decalcified maxilla of a rat comparable in age and size with those used in this experiment were made by using condensation silicone (Zetaplus; Zhermack, Badia Polesine RO, Italy).^23^ Preliminary casts with type IV stone (Durone; Dentsply Ind. Com., Rio de Janeiro, RJ, Brazil) were relieved with wax (2-mm thickness) on a previously determined extension of the palate. Custom trays were then fabricated with a colorless autopolymerizing acrylic resin (Vipi Flash; Vipi Ltda., Pirassununga, SP, Brazil) (Figure 1 A-B), and, after polymerization, the trays were trimmed and polished with a felt disc and polishing paste (Universal Polishing Paste for Resins and Metals; Ivoclar Vivadent Inc., Barueri, SP, Brazil). Palatal impressions of each rat were made with polyether (Impregum F; 3M do Brasil, Campinas, SP, Brazil) in the custom trays (Figure 1C).^23^ The type IV stone working casts obtained were waxed to a 3-mm thickness in the posterior region of the hard palate^23^ and 2-mm thickness in the buccal surface of molars (Figure 1D), as confirmed by the measurement with a periodontal probe (Hu-Friedy Brasil, Rio de Janeiro, RJ, Brazil).

The waxed casts were invested in metal flasks (OGP Prod. Odontol. Ltda., São Paulo, SP, Brazil) under pressure. The colorless denture base acrylic resin Vipi Cril (Vipi Ltda.) was manipulated according to the manufacturer instructions and packed into the mold invested within a flask and under pressure. The denture base acrylic resin was then subjected to the short cycle recommended by the manufacturer (73°C for 90 minutes plus 100°C for 30 minutes). After deflasking, the devices were finished with a mini-cut bur (NB364SE; Dhpro Rhadartrade Com. Imp. Peças Ltda.- Me., Paranaguá, PR, Brazil). The external surface of each device was then manually abraded with 100- and 200-grit silicon carbide papers (3M do Brasil) in a figure-of-eight motion,^24^ until they presented roughness values between 1 and 2 µm (Figure 1E).^8^ Finally, the devices were brushed with coconut soap and stored in distilled water at 37°C for 48 hours to release residual monomers and to simulate clinical conditions.^25^ Multiple individual impressions were made from six rats to obtain a total of 41 acrylic resin devices for different tests (N=20 for the sterilization tests, N=15 for the contamination tests, and N=6 for the stabilization test).

The acrylic resin devices were not purposely contaminated for the sterilization test. The devices (n=5) were assigned to one of the following conditions: 1. immersion in 200 mL of sterile distilled water and irradiation in a domestic microwave oven (MEF 41; Electrolux, Manaus, AM, Brazil) for 3 minutes (650 W);^11^ 2. exposure to ultraviolet light (2400 J cm^-2^, 60 Hz) for 3 minutes on each surface, both internal and external;^26^ or 3. immersion in an ultrasonic bath (Ultrasonic Cleaner 1440D; Odontobrás, Ribeirão Preto, SP, Brazil - 0.17 kW, 0.8 A, 42 kHz) in sterile distilled water for 20 minutes.^27^ As a negative control, five devices were not submitted to any sterilization method.

Swab samples were collected from the device surfaces, immersed in 2 mL of sterile phosphate-buffered saline (PBS), and vortexed for 30 seconds. Aliquots of 100 μL from this suspension were spread plated on Mueller-Hinton agar (Difco Zuidas, Amsterdam, Netherlands). The devices were then immersed in Falcon tubes containing 5 mL of brain-heart infusion (BHI) broth (Kasvi Imp. e Dist., Curitiba, PR, Brazil). Mueller-Hinton plates and BHI tubes were incubated for 48 hours at 37°C. Afterwards, the Petri dishes were visually evaluated. Then, 150-μL aliquots of each sample were transferred to a 96-well plate (TPP^®^) and 50 μL of 2,3,5-triphenyltetrazolium chloride (TTC, Vetec Química Fina Ltda., Duque de Caxias, RJ, Brazil) were added to each well for spectrophotometric analysis at 450 nm. All experiments were performed in triplicate.

### Palatal acrylic device contamination

The wild strain of *C. albicans* SC5314 was used in this study.^13^*C. albicans* was maintained in the yeast peptone glucose medium (YEPD; Clontech Laboratories Inc., Mountain View, CA) with 20% glycerol (Kasvi Imp. e Dist.) and frozen at −80°C until use.^13^ To prepare the inoculum, one loop of the unfrozen strain was plated on YEPD Agar (Clontech Laboratories Inc.) and incubated at 37°C for 48 hours.^13^ Then, one colony of *C. albicans* was removed from the plate, re-suspended in 20 mL of YEPD broth, and kept overnight at 37°C, yielding a preculture. For cell washing, the Falcon tubes were centrifuged (mod. 5804 R; Eppendorf do Brazil Ltda., São Paulo, SP, Brazil) at 4°C and 4000 rpm for 15 minutes, and the resulting supernatant was discarded. The pellet was rinsed with 5 mL of PBS and vortexed, centrifuged again as previously described, and the supernatant was discarded. This procedure was repeated twice.^13^ Then, the final pellet was resuspended in 20 mL of PBS and 10-fold dilutions were made. Aliquots of 25 μL from these dilutions were spread plated on Sabouraud Dextrose agar (Difco^®^) and incubated at 37°C for 48 hours for CFU/mL determination. The devices were contaminated with an inoculum of approximately 2.6 x 10^7^ CFU/mL.

For cell adhesion, each device was individually placed in one well in a 24-well culture plate (TPP) after microwave irradiation and immersed in 2 mL of inoculum. The culture plates were placed in a shaking incubator (Shaker – mod. 430/RDBP, Nova Ética, Vargem Grande Paulista, SP, Brazil) at a frequency of 75 rpm for 90 minutes at 37°C.^13^ Then, the non-adhered cells were removed by gently immersing the specimens in 2 mL of PBS. Finally, the devices were immersed in 2 mL of culture medium RPMI-1640 (Sigma-Aldrich Brasil Ltda., Duque de Caxias, SP, Brazil) and kept in the shaking incubator under the same conditions for 48 hours, with the culture medium being replaced every 24 hours.^13^

### Biofilm analyses

Additional palatal devices (N=15) were made, microwave irradiated, and contaminated with *C. albicans* to analyze the biofilm formed on their surfaces.

### CFU/mL determination

Microbiologic counting was performed on contaminated devices (n=5) to determine the viable burden of *C. albicans*. The specimens were placed in Falcon tubes containing 3 mL of PBS, sonicated for 2 minutes (0.17 kW, 0.8 A, 42 kHz), and vortexed for 30 seconds. Subsequently, 10-fold dilutions were made in sterile PBS, plated on Sabouraud Dextrose agar, and incubated at 37°C for 48 hours. The resulting colonies were visually counted and expressed as CFU/mL.

### Laser confocal microscopy

Other contaminated devices (n=5) were individually transferred to a 24-well culture plate containing 1 μL of SYTO-9 (Molecular Probes; Invitrogen Brasil Ltda., São Paulo, SP, Brazil) and 1 μL of propidium iodide (Sigma-Aldrich) diluted in 1 mL of PBS.^13^ The specimens were kept at rest in the solution for 20 minutes in the absence of light at 37°C to allow the diffusion of fluorochromes in the biofilm.^13^ Then, each device was mounted on a glass slab with immersion oil for biofilm analysis with a confocal laser scanning microscope (CLSM) (Leica LAS AF; Leica Microsystems GmbH, Wetzlar, Hesse, Germany) by using an objective lens (63×; maximum aperture of 1.15). The images were obtained with a software (Leica LAS AF) and standardized in four different fields. Four images were sequentially obtained from each section, with excitation of 488 nm for the SYTO 9 and 532 nm for propidium iodide. These four images were then combined so that viable and nonviable cells could be observed simultaneously in each field of each specimen.^13,28^ By using the software BioImageL v.2 (Faculty of Odontology, Malmö University, Malmö, Sweden), it was possible to obtain the total biovolume, viable biovolume, non-viable biovolume, percentage of cell viability, and the area covered by *C. albicans* biofilm.

### Scanning electron microscopy

For scanning electron microscopy (SEM) analysis, other acrylic resin devices (n=5) were fixed with 2.5% (v/w) glutaraldehyde (Sigma-Aldrich) for 150 minutes in 0.1 M PBS, pH 7.2. Then, the specimens were gently washed in triplicate with 2 mL of PBS, they underwent a graded dehydration series of ethanol (35, 50, 70, 80, 90, 95, 100, 100, 100% for 10 minutes each), and dried by using a graded series (50% and 100% for 10 minutes each) of hexamethyldisilazane (HMDS; Fluka Analytical, Munich, Bavaria, Germany). The devices were then kept for 2 days under a fume hood (Veco do Brasil, Ind. Com. de Equip. Ltda., Campinas, SP, Brazil) at 22°C. After drying, the devices were mounted on aluminum stubs (mod. IC-50 Ion Coater; Shimadzu, Kyoto, Japan) and sputter-coated with gold.^29^ Finally, the biofilm formed was analyzed with SEM (mod. SSX 550 Superscan; Shimadzu) at 10 kV and 100-second scan.

### Stabilization of the devices

**Figure 2:**
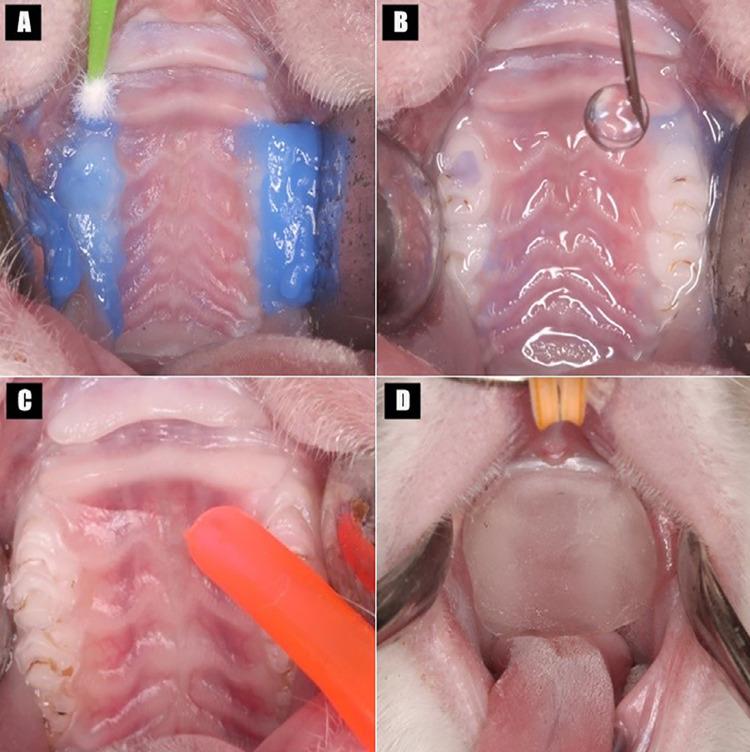
Cementation of devices. A-C: molars etched with phosphoric acid 37%, rinsed with distilled water, and then dried; D: device was cemented with a self-adhesive resin cement

Two devices were made and their fixation in the maxillary molars of the rats was tested with three different materials, which were randomly assigned: two autopolymerizing acrylic resins Vipi Flash and Duralay (Reliance Dental Mfg. Co., Alsip, IL) or a self-adhesive resin cement (RelyX U200; 3M ESPE, Campinas, SP, Brazil). The molar region of the devices was relieved to create space for cementation,^23^ except for those fixed with self-adhesive resin cement, which was allowed to self-cure without any exposure to curing light. Before the cementation of the devices with self-adhesive resin cement, the molars were etched with 37% phosphoric acid (Angelus Indústria de Produtos Odontológicos S/A, Londrina, PR, Brazil) for 30 seconds, rinsed with distilled water, and then dried (Figure 2A-D). These devices were maintained in position for up to 4 days to follow their stabilization.^30^

Three days before the placement of the palatal devices and during their use, the animals were fed a paste diet obtained by grinding the standard pellet diet (Presence; Socil Evialis Nutrição Animal Ind. Com. Ltda., São Paulo, SP, Brazil) mixed with tepid water.^23^

### Statistical analysis

Spectrophotometric data for comparing sterilization methods were first analyzed with the Shapiro-Wilk and Levene tests (IBM SPSS v19; SPSS Inc., IBM Company, Armonk, NY) (α=0.05). Then, spectrophotometric measurements (mAbs) were tested with one-way ANOVA followed by Tukey’s HSD post-hoc test (α=0.05) (IBM SPSS v19; SPSS Inc.). Sample power analysis was performed using GPower 3.1 (G*Power; Universität Düsseldorf, Düsseldorf, North Rhine-Westphalia, Germany).

## Results

### Animals

No significant weight loss was observed (data not shown), and no animals were lost during the experiments.

### Palatal acrylic resin device sterilization

For the number of specimens used (n=5), the study was adequately powered (96%; α=0.05) regarding the spectrophotometric analysis. Only microwave irradiation promoted sterilization of the palatal devices with (*P*=0.002) and without (*P*=0.001) the addition of TTC, as it did not present significant difference (*P*=1.000) compared with from the positive control (sterile BHI broth). Also, the MW group was the only one that did not present microbial growth in the culture plates. The UV group presented intermediate values with no statistically significant differences from any group (*P*>0.05). The US and NC groups had the highest values of contamination (*P*=0.015 and *P*=0.005 when compared to the sterile BHI broth, respectively).

### Biofilm analyses

**Figure 3:**
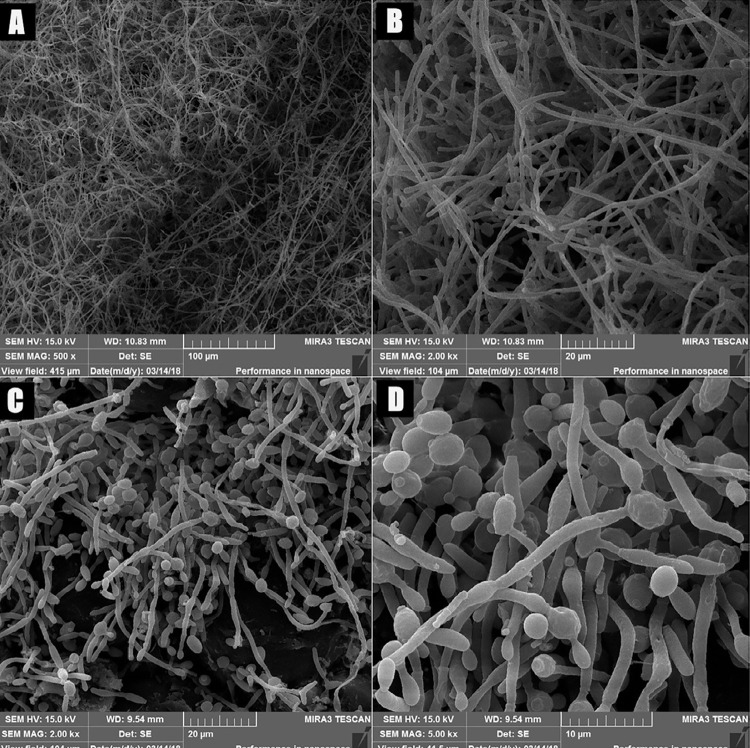
SEM photomicrographs showing *C. albicans* hyphae and blastospores on the internal surface of the contaminated devices. A: 500× magnification; B-C: 2.000× magnification; D: 5.000× magnification

**Figure 4:**
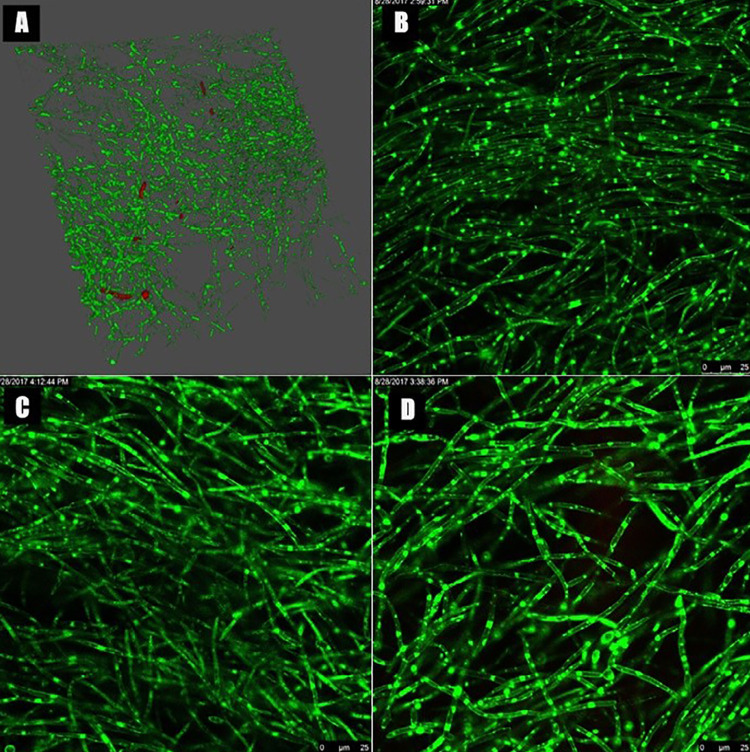
A: 3D digital image representing viable (green) and non-viable (red) *C. albicans* cells of the palatal acrylic devices after contamination. B-D: CLSM photomicrographs showing viable *C. albicans* hyphae on the external surface of contaminated devices. 63× magnification

The quantitative analysis of the biofilm formed on the contaminated devices was 1.2 × 10^6^CFU/mL on average. Colonies were counted at the 10^-3^dilution. The adhesion of hyphae and blastospores on the internal and external surfaces of the contaminated devices was confirmed by SEM and CLSM, respectively (Figures 3 and 4). Micrographs showed a mature biofilm developed on both surfaces. CLSM also confirmed the viability of the *Candida* cells, as viable hyphae and blastospores (3.6 x 10^7^ μm^3^of viable biovolume and 79.6 % of cell viability) were found in the biofilm formed on the surfaces of the palatal devices.

### Stabilization of the devices

Two days after cementation, devices fixed with Duralay acrylic resin were found detached in the oral cavity of the animals. Those fixed with Vipi Flash acrylic resin presented their central region covered with the material. However, the devices fixed with self-adhesive resin cement were still in position after four days.

## Discussion

Dental prostheses may act as a reservoir of microorganisms, allowing *C. albicans* to reinfect the oral mucosa and to contribute to the pathogenesis of denture stomatitis.^4^ Considering this hypothesis, a previously established biofilm formation protocol^13^ was used to contaminate the palatal devices. *C. albicans* viable hyphae and blastospores were found in the biofilm formed on the devices surfaces, as observed in the micrographs obtained by CLSM and SEM. The concentration of the inoculum used to contaminate the devices did not exceed the magnitude of 10^7^, as Chandra, et al.^31^ (2001) reported that more concentrated suspensions did not enhance biofilm formation on their specimens. The recovery of *C. albicans* from the contaminated devices (1.2 × 10^6^ CFU/mL) was approximately 10-fold lower than that initially inoculated (2.6 × 10^7^CFU/mL).

The acrylic resin devices should be previously sterilized to ensure that they would be contaminated only by *C. albicans*, the main etiological factor involved in the pathogenesis of denture stomatitis.^4^ In order to select the most appropriate method for sterilizing the devices, physical methods commonly used for dental materials were tested: microwave energy irradiation,^11^ exposure to UV light,^26^ and ultrasonic bath.^27^ Microwave energy irradiation at 650 W for 3 min was the only effective sterilization method. The use of microwave to decontaminate acrylic resin specimens and removable prostheses has been well described and it is recommended because of its low cost, safety, speed, and ease of handling, to mention a few of its advantages.^32^

The devices were fabricated with a colorless autopolymerizing acrylic resin to allow visibility during adaptation and fixation. Visibility at the time of fixation is essential, since any defect on the surface of the devices can compromise their adaptation and stabilization in the oral cavity. Norris, et al.^20^ (1985) incorporated miconazole nitrate to an autopolymerizing acrylic resin, aiming to develop a new treatment for denture stomatitis, and, as a consequence, the obtained devices were opaque. The opacity of the miconazole-supplemented acrylic resin hindered the authors’ observation of defects in the surfaces of devices, and nine appliances were lost during their experiments.

Different palatal device designs have been described in the literature. Some were fabricated without making an impression of the rat palates,^19,21^ compromising their fitting. Others have been fabricated with orthodontic wires and bands, which are placed on molars and/or incisors to serve as anchorage.^21,30^ Some authors developed a device prototype with fixed and removable portions, consisting of orthodontic wires and magnets,^22,33^ making its fabrication more complex. These elaborate designs and those covering the whole palate^18^ might irritate the animals and cause weight loss.^23^

The devices designed to cover only the posterior palatal mucosa and fixed on molars, as those fabricated in this study, can provide adequate comfort for the animals.^23^ The devices should remain in position for four consecutive days, the required time to induce denture stomatitis in rats according to Tobouti, et al.^30^ (2016). The self-adhesive resin cement provided the desired retention and it does not present the same disadvantages of autopolymerizing acrylic resins^23^ or composite resins^34^, such as the heating of the mucosa, either by exothermic reaction of the acrylic resin, or even by the temperature increase generated by the light curing units,^35^ which can compromise the analyses. Furthermore, it is not necessary to create a space at the molar region for fixing the devices with resin cement, unlike that required for attachment with acrylic or composite resin. Therefore, less material is necessary for cementation, ensuring that the internal portion of the device is in full contact with the palatal tissue.

The previous denture stomatitis models in rats inoculated *C. albicans* directly on the palate and/or on the internal portion of the palatal devices immediately before their fixation.^18,19,21,22,33^ Tobouti, et al.^30^ (2016) used a contamination protocol similar to the one used in our study, however, they reported that only the internal portion of the devices was contaminated and found no signs of candidiasis on the animal tongues. One of the main advantages of the proposed device design is the possibility of infecting the palate and tongue simultaneously, as the devices are totally immersed in the inoculum during their contamination and a mature *C. albicans* biofilm can be developed on both internal and external surfaces.

There were no significant changes in the animal weight, nor deaths resulting from the use of devices or consumption of the pasty diet, indicating that this protocol is safe. Considering the previous devices design limitations and all the accomplished improvements (i.e., reproducibility of methods, efficient sterilization and contamination protocol, adequate visibility during device adaptation, and good and sufficiently long stabilization in the oral cavity), the proposed device design is expected to be a valuable tool in the development of denture stomatitis in rats, even with the many manufacturing steps required to create the device. Further studies are being conducted to evaluate the effectiveness of this palatal device to induce denture stomatitis in Wistar rats.
